# Prevalence of Pelvic Floor Dysfunction in Women with Cervical Cancer: A Cross-Sectional Study

**DOI:** 10.1007/s00192-026-06582-y

**Published:** 2026-03-24

**Authors:** Mariana Basilio Andrade, Taynara Louisi Pilger, Francisco José Candido dos Reis

**Affiliations:** Department of Gynecology and Obstetrics. Ribeirão Preto Medical School, University of Sao Paulo, Av. Bandeirantes, 3900, Ribeirão Preto, SP 14049-900 Brazil

**Keywords:** Pelvic floor disorders, Pre-treatment assessment, Urinary incontinence, Uterine cervical neoplasms

## Abstract

**Introduction and Hypothesis:**

Pelvic floor dysfunction is frequently associated with cervical cancer treatment; however, their baseline prevalence at diagnosis is unknown. This study aimed to investigate pelvic floor dysfunction in newly diagnosed cervical cancer, before oncologic treatment.

**Methods:**

Cross-sectional analysis of women newly diagnosed with cervical cancer at a single institution in Brazil. Participants answered a structured interview and the FSFI questionnaire and underwent a pelvic floor structured physical examination. Analyses were performed in RStudio.

**Results:**

A total of 48 women were included, 19/48 (39.6%) with early-stage and 29/48 (60.4%) with locally advanced cervical cancer. Groups were similar in sociodemographic and reproductive characteristics. Sexual dysfunction was observed in 27/48 (56%) participants. Pelvic floor muscle strength and endurance were comparable between groups, with a median Oxford Scale score of 3 (2–4) and endurance of 5 s. Urinary incontinence was present in 25/48 (52%) participants, while fecal incontinence occurred only in 1/48 (5.3%).

**Conclusions:**

Pelvic floor dysfunction is common in women newly diagnosed with cervical cancer. Early recognition is crucial to differentiate pre-existing from treatment-induced dysfunction and to guide prehabilitation and rehabilitation strategies that may improve quality of life.

## Introduction

Cervical cancer is among the most frequently diagnosed neoplasms in the world, constituting the fourth leading cause of cancer mortality among women [1]. Approximately 90% of cases occur in developing countries [2]. The clinical management of cervical cancer requires a multidisciplinary approach, involving gynecologists, oncologists, radiotherapists, nurses, physiotherapists, psychologists, and other health professionals. This integration is crucial for developing individualized therapeutic plans that optimize diagnosis, treatment planning, complication management, and promote patients’ quality of life [3]. Treatment should be adapted to the clinical characteristics and specific needs of each woman, varying according to the stage of the disease. In the early stages, surgery represents the main therapeutic modality, while in locally advanced cases, chemoradiation is the recommended approach [4, 5].

The treatment of cervical cancer is frequently associated with pelvic floor dysfunction. The prevalence of female sexual dysfunction, urinary incontinence (UI), and fecal incontinence (FI) in women after treatment has been reported as 45.0%, 34.1%, and 11.1%, respectively [6]. However, pelvic floor dysfunction is multifactorial. Associated factors included advanced age, higher body mass index (BMI), menopause, pregnancies and vaginal deliveries, instrumental deliveries, episiotomy, perineal lacerations, fetal macrosomia, and gastrointestinal or urological pathologies [7]. In a sample of 1446 healthy women in Spain, 55.8% reported UI, 10.4% FI, 14.0% symptomatic uterine prolapse, and 18.7% pelvic pain. The mean impact of these conditions, assessed using the PFDI-20, was 14.33 points for prolapse, 15.69 for colorectal–anal symptoms, 22.21 for urinary symptoms, and 52.23 for the total score [7].

However, there is still a lack of evidence regarding pelvic floor dysfunction present before the start of cancer treatment, as these conditions remain poorly recognized, often underdiagnosed, and surrounded by imprecise epidemiological estimates [8]. A baseline urinary incontinence rate of approximately 35.3% and a fecal incontinence rate of 5.9% were observed in the cervical cancer subgroup of women evaluated for suspected gynecological malignancy prior to surgical intervention, with these symptoms reported via a survey instrument [9]. The limited knowledge about the prevalence and severity of these alterations in newly diagnosed women hampers accurate estimation of the burden attributable to oncological treatment.

The present study aims to evaluate the prevalence and characterize pelvic floor dysfunction in women newly diagnosed with invasive malignant cervical neoplasms, including changes in pelvic muscle strength and endurance, sexual dysfunction, and urinary and fecal incontinence. Estimating the presence of these conditions before the start of treatment is essential to accurately identify the incidence of pelvic floor dysfunction resulting from cancer therapies, allowing differentiation between pre-existing alterations and those caused by treatment.

## Methods

### Study Design

A cross-sectional study was conducted at a tertiary hospital. Data collection spanned from February 2023 to September 2025. The study protocol was approved by the institutional research ethics committee, and all participants provided written informed consent.

### Population and Eligibility Criteria

Eligible participants were women aged ≥ 18 years with a histopathological diagnosis of invasive cervical carcinoma. Diagnosis was confirmed by biopsy, and disease staging followed clinical and radiological criteria based on the International Federation of Gynecology and Obstetrics (FIGO) classification [10].

Exclusion criteria were previous cancer treatment for cervical cancer, current pregnancy, neurological impairment preventing communication, metastatic disease eligible exclusively for palliative care, and inability to provide informed consent.

### Recruitment and Sampling

Participants were consecutively recruited during consultations and examinations before undergoing cancer treatment. To ensure transparency, a flowchart was developed detailing the number of eligible patients, refusals, and reasons for exclusion. Participants were subdivided into two groups according to the FIGO staging of invasive cervical carcinoma and the corresponding standard treatment approach. The early-stage group included women with disease up to FIGO stage IB2, who are typically candidates for primary surgical management, whereas the locally advanced group comprised stages IB3 to IVA, which usually require concurrent chemoradiation as the standard treatment.

### Sample Size

This was a cross-sectional baseline analysis nested within a prospective cohort. Therefore, all consecutive eligible women evaluated during the data collection period were included. As the study aimed to describe the prevalence of pelvic floor dysfunction in a real-world clinical population rather than to test a predefined hypothesis, a formal sample size calculation was not applied. This approach is consistent with observational designs in which the sample reflects all available cases within the recruitment window, ensuring representativeness of the population treated at our institution.

### Sociodemographic Variables

Sociodemographic and clinical information were recorded, including age, BMI, self-identified race/color category (e.g., White, Black, Brown, Asian), sexual orientation, marital status, educational level, reproductive status, obstetric history, type of delivery, lifestyle (smoking, alcohol consumption, and physical activity), sexual activity, menopausal status, tumor stage, and histological type. Data were collected through a structured interview conducted with each participant immediately after signing the informed consent form.

### Assessed Outcomes

All pelvic floor assessments were performed by trained researchers with expertise in pelvic health, using standardized muscular and gynecological examination protocols to minimize inter-examiner variability. Pelvic floor dysfunction was evaluated using an integrated approach, combining a detailed clinical examination with structured self-report instruments. PFM strength and endurance were assessed by vaginal bidigital palpation. Strength was measured using the Modified Oxford Scale (0–5 points), where 0 indicates absence of contraction and 5 indicates strong contractions with positive movement toward the pubic symphysis. Endurance was measured as the time, in seconds, during which the participant was able to maintain a sustained submaximal voluntary contraction, estimating muscular resistance and the ability to maintain contraction over time [11, 12].

Sexual dysfunction was assessed using the Female Sexual Function Index (FSFI), a 19-item questionnaire evaluating the domains of desire, arousal, lubrication, orgasm, satisfaction, and pain during sexual activity in the 4 weeks preceding the assessment [13, 14]. The cutoff score for classifying female sexual dysfunction using the FSFI was originally established at 26.55 by Wiegel, Meston, and Rosen (2005), based on ROC curve analysis comparing women with and without clinical sexual dysfunction. This threshold provided the best balance between sensitivity and specificity and was therefore adopted as the reference for identifying women with probable sexual dysfunction. In the Brazilian context, however, the cross-cultural validation of the FSFI into Portuguese proposed a slightly higher cutoff score (27.5) to better reflect local population characteristics [15]. Thus, the present study adopted the cutoff score of 27.5, as recommended in the validated Portuguese version, for the classification of sexual dysfunction.

### Statistical Analysis

Continuous variables were presented as medians and interquartile ranges, and categorical variables as frequencies and percentages. For group comparisons, appropriate bivariate tests were used: the Mann–Whitney test for continuous variables and the chi-square or Fisher’s exact test for categorical variables. The significance level adopted was *p* < 0.05. All analyses were performed using R Studio software.

## Results

**Fig. 1:**
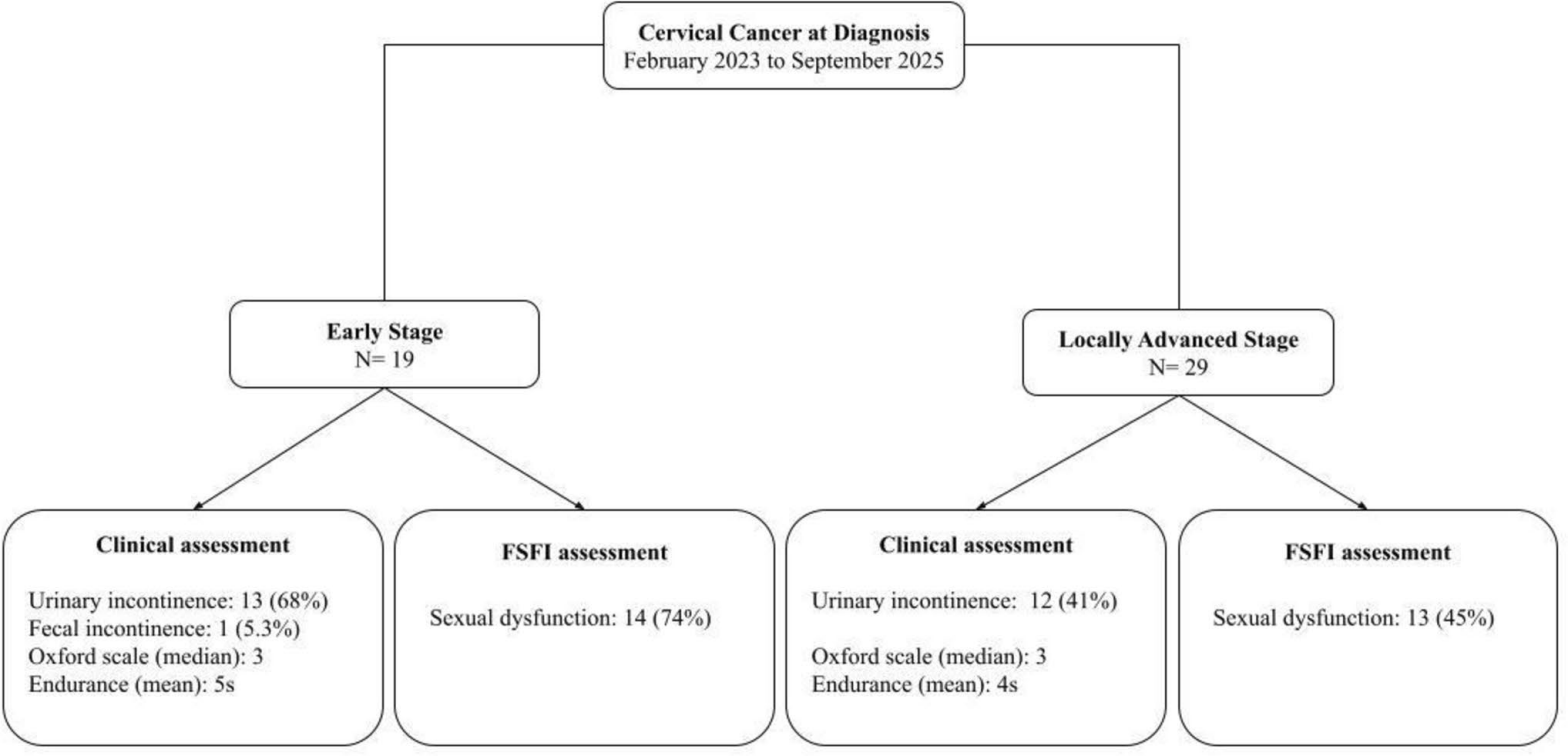
Flow chart of participants

A total of 48 participants were included, 19 with early-stage cervical cancer (ESCC) and 29 with locally advanced cervical cancer (LACC). The patient flow is shown in Fig. 1.

### Participant Characteristics

The characteristics of the participants are presented in Table 1. No significant differences were observed between the groups regarding sociodemographic variables, indicating homogeneity between the groups. The median age of the participants was 41 years (interquartile range [IQR] 34–49). Most women were white (29/48; 60%), heterosexual (47/48; 98%), married (16/48; 33%), and had an intermediate educational level. The majority were of reproductive age (34/48; 71%), with a median parity of three (IQR 2–4) and a median of one cesarean section (IQR 1–2). The median BMI was 28.9 kg/m^2^ (IQR 24.8–32.4). When stratified by stage, the early-stage group and advanced-stage group had similar median ages (40 years [IQR 33–57] and 42 years [IQR 35–48], respectively). The racial distribution was 47% white in the early-stage group (9/19) and 69% white in the advanced-stage group (20/29). Sexual orientation was predominantly heterosexual, 95% (18/19) in the early group and 100% (29/29) in the advanced group. Reproductive age was noted for 63% (12/19) of the early group and 76% (22/29) of the advanced group.

**Table 1:** Participants characteristics by tumor stage group

Characteristic	Overall *N* = 48^1^	Early *N* = 19^1^	Advanced *N* = 29^1^	*p* value^2^
Age	41 (34, 49)	40 (33, 57)	42 (35, 48)	0.8
Race				0.4
White	29 (60%)	9 (47%)	20 (69%)	
Asian	1 (2.1%)	1 (5.3%)	0 (0%)	
Brown	13 (27%)	7 (37%)	6 (21%)	
Black	4 (8.3%)	2 (11%)	2 (6.9%)	
Unknown/not reported	1 (2.1%)	0 (0%)	1 (3.4%)	
BMI	28.9 (24.8, 32.4)	30.8 (24.2, 32.4)	28.8 (24.8, 32.3)	0.9
Sexual orientation				0.4
Heterosexual	47 (98%)	18 (95%)	29 (100%)	
Homosexual	0 (0%)	0 (0%)	0 (0%)	
Bisexual	1 (2.1%)	1 (5.3%)	0 (0%)	
Marital status				0.2
Single	12 (25%)	5 (26%)	7 (24%)	
Married	16 (33%)	9 (47%)	7 (24%)	
Stable union	10 (21%)	1 (5.3%)	9 (31%)	
Divorced	6 (13%)	3 (16%)	3 (10%)	
Widower	4 (8.3%)	1 (5.3%)	3 (10%)	
Educational level				0.5
Illiterate	2 (4.2%)	0 (0%)	2 (6.9%)	
Incomplete elementary school	15 (31%)	5 (26%)	10 (34%)	
Complete elementary education	5 (10%)	1 (5.3%)	4 (14%)	
Incomplete high school	3 (6.3%)	1 (5.3%)	2 (6.9%)	
Complete high school	18 (38%)	8 (42%)	10 (34%)	
Incomplete higher education	2 (4.2%)	2 (11%)	0 (0%)	
Graduate	3 (6.3%)	2 (11%)	1 (3.4%)	
Reproductive status				0.3
Reproductive age	34 (71%)	12 (63%)	22 (76%)	
Post menopause	14 (29%)	7 (37%)	7 (24%)	
Parity	3 (2, 4)	3 (2, 4)	4 (2, 5)	0.11
Cesareans	1 (1, 2)	1 (1, 2)	1 (1, 2)	0.8
Tobacco use	10 (21%)	1 (5.3%)	9 (31%)	0.065
Alcohol use	6 (13%)	1 (5.3%)	5 (17%)	0.4
Physical activity	7 (15%)	3 (16%)	4 (14%)	> 0.9
Sexual activity	27 (56%)	11 (58%)	16 (55%)	0.9
Tumor type				0.6
Squamous carcinoma	42 (88%)	16 (84%)	26 (90%)	
Adenocarcinoma	5 (10%)	3 (16%)	2 (6.9%)	
Neuroendocrine carcinoma	1 (2.1%)	0 (0%)	1 (3.4%)	

^1^Median (Q1, Q3); *N* (%)

^2^Wilcoxon rank sum exact test; Fisher’s exact test; Wilcoxon rank sum test; Pearson’s chi-squared test

Tobacco use was reported by 5.3% (1/19) of early-stage and 31% (9/29) of advanced-stage participants, and alcohol consumption by 5.3% (1/19) and 17% (5/29), respectively. Physical activity was reported by 16% (3/19) and 14% (4/29), respectively. The predominant histological tumor type was squamous cell carcinoma, accounting for 84% (16/19) of early-stage and 90% (26/29) of advanced-stage cases.

Tumor stages within the overall cohort were distributed as follows: IA1 (6/48; 13%), IA2 (2/48; 4.2%), IB1 (7/48; 15%), IB2 (4/48; 8.3%), IB3 (2/48; 4.2%), IIA2 (1/48; 2.1%), IIB (8/48; 17%), IIIA (1/48; 2.1%), IIIB (5/48; 10%), IIIC1 (8/48; 17%), IIIC2 (2/48; 4.2%), and IVA (2/48; 4.2%).

### Outcomes

Data on the prevalence of pelvic floor dysfunction according to tumor stage are presented in Table 2.

**Table 2:** Outcomes according to tumor stage groups

Characteristic	Overall *N* = 48	Early *N* = 19	Advanced *N* = 29	*p* value
Sexual dysfunction (FSFI < 27.5)^1^	27 (56%)	14 (74%)	13 (45%)	0.049^3^
Oxford Scale reviewed^2^	3 (2, 4)	3 (2, 4)	3 (1, 4)	0.6^4^
Endurance (sustaining time,s)^2^	5 (2)	5 (3)	4 (2)	0.2^4^
Urinary incontinence^1^	25 (52%)	13 (68%)	12 (41%)	0.067^3^
Fecal incontinence^1^	1 (2.1%)	1 (5.3%)	0 (0%)	0.4^3^

^1^*N* (%)

^2^Median (Q1, Q3); mean (SD)

^3^Pearson’s chi-squared test

^4^Wilcoxon rank sum test

### Sexual Function

**Fig. 2:**
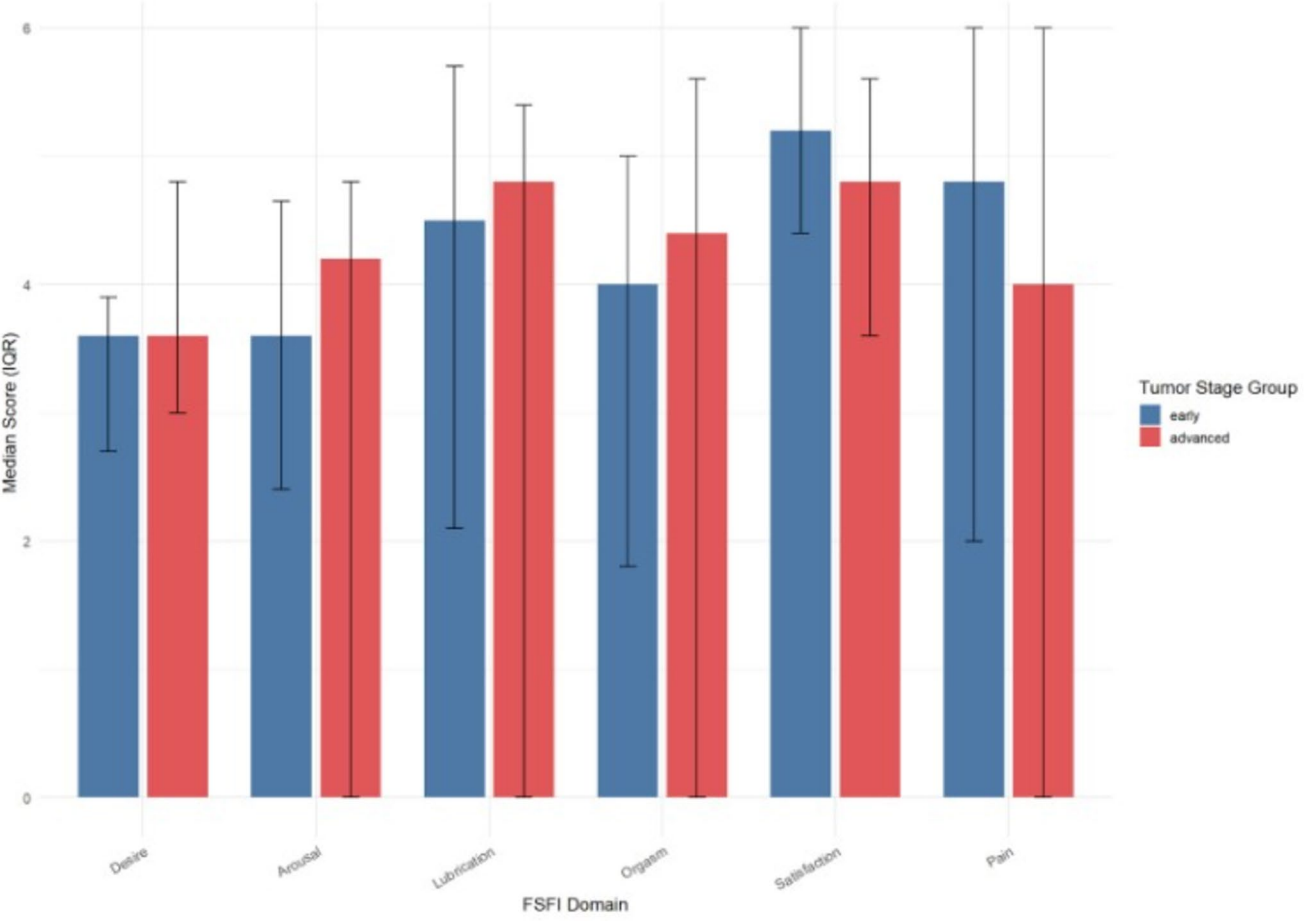
Median scores of the FSFI domains by tumor stage group. Bars represent the median and interquartile range of scores in the domains of desire, arousal, lubrication, orgasm, satisfaction, and pain, comparing the early stage (blue) and the locally advanced stage (red)

Overall, 56% (27/48) of participants were sexually active. Sexual dysfunction, assessed by the FSFI, was observed in a substantial proportion of participants, occurring in 74% (14/48) of the early-stage group and 45% (13/48) of the advanced-stage group (*p* = 0.049). Analysis of the FSFI domains revealed similar scores between groups, with a broad overlap of interquartile ranges (Fig. 2).

### Pelvic Floor Strength and Endurance

No significant differences were observed between groups in any of the evaluated parameters. The median Oxford Scale score for the total sample was 3 (2–4), without a difference between the groups. The mean endurance time of voluntary contraction was 5 s in both groups. Endurance could not be measured in one participant from the ESCC group due to the absence of voluntary contraction.

### Urinary and Fecal Incontinence

Overall, urinary incontinence was reported by 52% (25/48) of participants, while fecal incontinence was reported by 2% (1/48). No statistically significant differences were observed between tumor stage groups.

## Discussion

In this study, we found a high prevalence of pelvic floor dysfunction among women newly diagnosed with cervical cancer. The similar distribution of dysfunction across early and locally advanced stages indicates that these impairments are likely driven by shared underlying risk factors rather than by the disease stage itself. Education level, economic status, early sexual debut/early sexual initiation, age at first pregnancy, and parity are cofactors to HPV infection in cervical cancer development and progression [16].

Among the factors associated with pelvic floor dysfunction observed in this study, BMI deserves particular attention. Overall, 57% of participants had a BMI in the overweight or obese range, highlighting that a substantial proportion of the sample was above the normal weight. This high prevalence of overweight and obesity in our cohort is critical, as it may increase the generalizability of our findings given the concurrent global rise in average BMI and the well-documented association between BMI and pelvic floor disorders [17]. The prevalence of urinary incontinence in the general female population ranges broadly from 25 to 45% in many international studies, while baseline rates among women with gynecologic malignancy are often reported to be higher, ranging from 40 to 59% prior to definitive cancer treatment [9, 18, 19]. Studies have demonstrated that being overweight increases the risk of urinary incontinence by approximately 35%, and obesity nearly doubles this risk compared to women with a normal weight [20, 21].

In the present sample, urinary incontinence was reported by a significant proportion of participants, reinforcing that this is a frequent co-existing condition in women with cervical cancer. Similar results were described by White et al., who identified a prevalence of UI of up to 53.9% in women with endometrial or uterine cancer, being considered a “major problem” by approximately 20% of the participants [22]. These data show that UI represents a clinically relevant symptom in oncologic patients and deserves attention even before the beginning of treatment. In addition, it is also possible that the BMI variable contributed to the strength levels observed. Studies show that obesity is associated with reduced PFM strength, both in phasic and tonic contractions, and that increased intra-abdominal pressure resulting from excess weight can lead to overload and weakening of this musculature [23].

The analysis of pelvic floor muscle strength revealed a median grade of 3 on the Oxford Scale in both groups, indicating moderate contraction intensity. This level is consistent with reference values reported in the literature, where most women exhibit strength grades of three or below, reflecting a generally moderate functional range. Studies with similar populations have shown that approximately one-third of women reach grade 3 on the Oxford Scale, suggesting that the values observed in the present study are within the expected range for the general female population [24, 25]. However, it should be considered that the Oxford Scale, although widely used, is a subjective method for assessing pelvic floor muscle strength, dependent on the examiner’s experience and sensitivity, which may limit its accuracy in detecting small variations in muscle pressure. The bidigital palpation technique, on which the scale is based, is recognized as less sensitive when compared to instrumental methods, as it relies on clinical judgment and tactile perception [26]. Thus, although the results indicate moderate strength, these values should be interpreted with caution, since small differences between examiners or clinical conditions may influence the measurements.

Finally, the high prevalence of sexual dysfunction observed in our participants reinforces the negative influence of a cancer diagnosis on sexual function, regardless of disease progression. This finding is consistent with the results of Qian et al., who reported a sexual dysfunction prevalence of approximately 80% among women with cervical cancer, showing a clear difference compared to the non-cancer population [27]. In view of this, the systematic inclusion of sexual function assessment from the moment of diagnosis, as well as the implementation of psychosocial support and sexual education strategies for women with gynecologic neoplasms, becomes essential to mitigate its impact on quality of life.

Given the high baseline prevalence of pelvic floor dysfunction observed in women newly diagnosed with cervical cancer, the most critical future direction is the longitudinal assessment of these symptoms following treatment. We are planning a follow-up study, which will utilize our existing prospective cohort to evaluate changes in urinary, fecal, and sexual function after surgical and/or chemoradiation therapy. This approach will provide valuable, time-dependent insights into the impact of oncologic treatment on pelvic floor health, identify risk factors for worsening dysfunction, and ultimately inform the development of evidence-based, early interventions to enhance quality of life in this survivor population.

Our study has several key strengths. Data were collected by trained researchers using standardized protocols for both muscular and gynecological assessments, minimizing inter-examiner variability and ensuring methodological rigor. The use of validated instruments, such as the FSFI, and an integrated approach to evaluating pelvic floor dysfunction ensured a comprehensive clinical assessment. Furthermore, the inclusion of all consecutive eligible participants at our tertiary hospital enhances the representativeness of the cohort treated at our institution, and the division of participants according to disease stage allowed for clinically relevant comparisons between early and locally advanced cervical cancer.

However, some limitations should be acknowledged. First, the cross-sectional design is a fundamental constraint, preventing us from making causal inferences about the relationship between cervical cancer and pelvic floor dysfunction. Second, a relatively small sample size, the lack of a formal sample size calculation, and the recruitment from a single tertiary hospital may limit the generalizability of our findings and potentially introduce selection bias. Third, the assessment of pelvic floor muscle strength relied on the Oxford Scale, a subjective measure that is dependent on examiner experience and may lack the sensitivity to detect subtle functional differences. Finally, some relevant confounding factors, such as a history of prior pelvic floor training or pre-existing comorbidities, were not systematically accounted for.

## Conclusion

Pelvic floor dysfunction is prevalent among women newly diagnosed with cervical cancer across all stages. Establishing the baseline status of this dysfunction is essential to differentiate between pre-existing impairments and those that are treatment-induced, thus allowing for accurate attribution of morbidity. Early identification of deficits informs patient counseling and permits the immediate implementation of a triage strategy for pelvic floor health. This approach includes education, behavioral modifications, and, where appropriate, referral for preparatory interventions before definitive oncological treatment. This study, therefore, underscores the need for integrating routine pelvic floor assessment into the immediate pre-treatment care pathway, which in turn sets the stage for accurate prospective follow-up and effective post-treatment rehabilitation.

## Data Availability

The anonymized data supporting the findings of this study are available from the corresponding author upon reasonable request.

## Abbreviations

UI: Urinary incontinence
FI: Fecal incontinence
PFM: Pelvic floor muscle
FSFI: Female Sexual Function Index
ESCC: Early-stage cervical cancer
LACC: Locally advanced cervical cancer
